# Hepatoblastoma in Adult: Review of the Literature

**DOI:** 10.4021/jocmr2009.01.1222

**Published:** 2009-03-24

**Authors:** Ming Hua Zheng, Lei Zhang, Dian Na Gu, Hong Qi Shi, Qi Qiang Zeng, Yong Ping Chen

**Affiliations:** a Department of Infection and Liver Diseases, The First Affiliated Hospital of Wenzhou Medical College, Wenzhou, China; b Department of General Surgery, The First Affiliated Hospital of Wenzhou Medical College, Wenzhou, China

## Abstract

This study is to review and retrieve data on adult hepatoblastoma (HB) from English literatures in order to gain a better understanding of this disease. We performed Medline, PubMed (from January 1966 to February 2008), and library searches (National Science and Technology Library, Beijing, China, and Wenzhou Medical College Library, from January 1980 to February 2008) using the key words hepatoblastoma in adult, hepatic tumor, hepatoblastoma and adult. Previously reported HB cases were collected and published reviews were also examined. Fifteen cases that met the search criteria were selected. Review of the cases revealed a slight female preponderance. The patients' age ranged from 17 to 82, with median age of 70 for male and 27 for female. The survival time ranged from two weeks to 38 months, and the median survival time was 6 months. In the articles reviewed, HB presented with non-specific initial symptoms, and the diagnosis was not identified until the tumor biopsy after operation or autopsy. Completely surgical resection is still the major treatment for patients with HB and is considered as the only chance of a better prognosis. Due to the rareness of HB in adults, the choice of treatment should be radical resection if possible, and combined with chemotherapy as adopted in children. HB in the adult is extremely rare and the pre-operative diagnosis is often overlooked. The prognosis is so poor that the awareness of the condition in the differential diagnosis in liver tumors could be beneficial.

**Key words:**

Hepatoblastoma; Adult; Diagnosis; Therapy

## Introduction

Hepatoblastoma (HB) is a rare malignant tumor of the liver, which comprises over two-thirds of the malignant tumors of the liver in children [1], with most occurring before the age of 5 [2]. It accounts about 1-4% of all primary malignancy in children [3]. Most of these tumors arise in the embryo, hence it seems to be unusual that HB occur in adults and are an exceptional cause of primary malignant liver tumor in adult patients [4]. Although the existence of HB in adult patients has been refuted by some authors, approximately 40 adult cases of HB have been reported [5-7], with non-specific initial symptoms and difficulty in discerning irregularities in the laboratory studies of the patients. Consequently, the diagnosis is often overlooked, and patients may present at the late stages of the disease at risk of increased mortality.

## Literature retrieval

We performed Medline, PubMed (from January 1966 to February 2008), and Library searches (National Science and Technology Library, Beijing, China, and Wenzhou Medical College Library, from January 1980 to February 2008) using the following keywords, hepatoblastoma in the adult, hepatic tumor, hepatoblastoma and adult. In addition, references of the articles were obtained and previous reviews were also examined. The literature searches were limited to the English language literature. All patients listed in Table 1 are over 17 years old, the cases associated with other pathologies, such as hepatocellular carcinoma (HCC), were excluded.

We retrieved 15 cases based on the search criteria. Table 1 lists 14 articles that met the inclusion criteria [3-5, 7-17]. There were 7 cases in case reports [3, 5, 7-9, 14, 17]; other 6 cases were in the review literatures [4, 10-12, 15, 16] and in one case series [13].

**Table 1:** Summary of hepatoblastoma in adults

Ref. No.	Sex/Age (yr)	Location	Size (cm)	Histology	Procedure	Survival (Mon)	POD	MET
8	M/78	L	19×6×1	Mixed	SR	1	Ab	Y
9	F/19	R	19	Mixed	SR	NR	NR	NR
10	F/27	R	25	Mixed	SR	18	ALA	N
11	M/22	L	6.5×6×6	Embryonic	SR+TAE+CT	9	NR	Y
12	M/82	L+R	Child head size	Mixed	CT+TAE	6	HCC	N
13	M/73	NR	18×18×10	Mixed	NR	NR	NR	NR
13	F/35	NR	NR	Epithelial	NR	NR	NR	NR
14	F/22	Medial part	14×13×13	Epithelial	SR	38	NR	N
4	F/51	L	11×7	Mixed	SR	2	HCC	NR
7	M/67	R	8×6	Mixed	SR	0.5	NR	N
5	F/78	R	23	Mixed	SR	2	NR	N
15	F/52	L+R	22×16×15, 2.5	Mixed	SR+RFA	6	HCC	NR
16	F/19	R	14×12×10	Mixed	SR+CT	6	NR	NR
3	F/17	R	11.9×10.4	Epithelial	CT	NR	NR	NR
17	M/34	R	17×11×9	Mixed	SR	3	NR	N

M: Male; F: Female; L: Left lobe; R: Right lobe; Y: Yes; N: No; NR: No report; SR: Surgical resection; CT: Chemotherapy; TAE: Transcatheter arterial embolization; RFA: Radio-frequency ablation; POD: Pre-operative diagnosis; Ab: Abdominal aortic aneurysm; ALA: Amoebic liver abscess; HCC: hepatocellular carcinoma; MET: Metastasis.

## Discussion

HB is the most common primary malignant liver neoplasm in children [1, 18]. Approximately 90% of the cases occur in patients under 5 years of age and two thirds of the cases occur in the first 2 years of life [2, 19]. Boys are affected twice than girls [20]. HB in adolescent and young adults is extremely rare and the prognosis is much worse than the HB in childhood, because they are usually diagnosed late [4, 16]. Review of the studies on HB in the adults, revealed a slight female preponderance (9 female versus 6 male). The age of the patients reported in the literature ranged from 17 to 82, with a median age of 70 years for male and 27 years for female.

The etiology of HB has been elusive. Present investigations of the cytogenetic and molecular genetic abnormalities in HB revealed involvement of chromosomal loci on 1q, 2 (or 2q), 4q, 8 (or 8q), and 20 [21]. Loss of heterozygosity imprinting at locus 11p 15.5 also suggests a common genetic basis for HB [21]. The detection of nuclear β-catenin accumulation implies an oncogene alteration of the wnt/β-catenin pathway. Furthermore, nuclear p53 accumulation indicates that p53 mutation is also involved in the molecular pathogenesis of the malignancy [22]. Based on embryological theory, it is believed that HB arises from a hepatic blastema. However, this hypothesis seems to be inapplicable to adult HB. Four patients [5, 8, 12, 13] in the literature were more than 70 years old. The persistence of primitive hepatic blastema for such a long period seems unlikely. Furthermore, the presence of cirrhosis in liver with HB is not seen in children. However, cirrhosis of the liver has been seen in association with adult HB in 30% of cases [5, 6]. This would imply that these tumors may have a different pathogenetic pathway in adults compared to children.

The histological classification of HB was originally proposed by Ishak and Glunz [23], and has now gained wide acceptance. Among the cases summarized in Table 1, the mixed type (11/15) was predominated compared to the epithelial type (4/15). The tumors can be unifocal or multifocal, most occur as single mass (12/15) either in right lobe (7/12) of liver or in left lobe (3/12), or both of the two lobes (2/12). There is only one case reported as multifocal (1/15). The sizes of the masses ranged from 2.5 to 25 cm. The usual presentation is failure to thrive, loss of weight and a rapidly enlarging upper abdominal mass. Some presented with pain, fever and vomiting.

The initial diagnosis of HB is mainly based on imaging. Proper diagnosis, staging and treatment of HB require accurate imaging studies. Ultrasound (US) is a non-invasive modality that is particularly useful in the evaluation of infants. HB is seen as a hyperechoic, solid, intrahepatic mass on US [24]. Other standard investigations include computed tomography (CT), magnetic resonance imaging (MRI), serum AFP and β-HCG. However, the final diagnosis relies on tumor biopsy. While in the adult, the morbidity of HB is extremely rare and the initial symptoms are non-specific so that the diagnosis is often overlooked. Furthermore, it is also difficult to make a pathologic diagnosis of adult HB, since there are several similar types of tumor such as hepatic teratoma, carcinosarcoma, malignant mesenchymal tumor and HCC with sarcomatous changes and hepatoblastomatous lesions [12]. In almost all of the cases summarized in Table 1, the diagnosis of HB was not made until the tumor biopsy or autopsy. The pre-operative diagnosis (POD) of three cases were HCC [4, 12, 15] and two cases were abdominal aortic aneurysm and hepatic amoebic abscess [8, 10] respectively.

The complete surgical resection is the cornerstone of treatment for patients with HB and is the only chance of an optimal clinical outcome. Despite this, the improvements in survival that have occurred over the last three decades have been a function of standardized chemotherapy regimens that reduce tumor size and enable complete tumor excision, even permitting cure in the presence of initially unresectable or metastatic disease [25]. Chemotherapy has been proven effective in both an adjuvant and neoadjuvant treatment and can shrink tumors. It makes them less prone to bleed and delineates the tumor from the surrounding normal parenchyma and vascular structures so as to facilitate the resections. HB is sensitive to such chemotherapy drugs as doxorubicin, cisplatin, vincristine, 5-FU and cyclophosphamide [26]. Furthermore, liver transplantation has recently been associated with significant success in the treatment of children with unresectable hepatic tumors. Post-transplant survival rates as high as 80% have been reported for children with HB [27]. The current 5-year survival rate in children is 75% compared with a 5-year survival rate of 35% almost 30 years ago [18, 28]. Because lack of experience of HB in adult patients, it is reasonable to select radical resection and chemotherapy for HB in adults. Of the 15 cases, 9 cases were treated with surgical resection, 2 with surgery and postoperative chemotherapy, and one with transcatheter arterial embolization (TAE).

Despite the progress of therapy, 20%-30% of HB patients have a fatal outcome. Prognostic factors are needed for better therapy planning in these patients [29]. The prognosis is poor in multiple lobes involvement, embryonic type, decreased P27 gene expression, multifocal dissemination, AFP level lower than 100 or higher than 100000 ng/ml [3, 30], and the RASSF1A methylation [29]. In our review of the literature, we found there were 5 cases (33.3%) with AFP increasing [4, 5, 11, 14, 17]. The survival time ranged from two weeks to 38 months, and the median survival time was 6 months, this was much worse compared with childhood patients.

With the low incidence and non-specific initial symptoms, HB in the adult presents a diagnostic challenge, demanding a high index of suspicion and a thorough evaluation. Because the prognosis could be improved with early detection, diagnosis and treatment, it is important for clinicians to be aware of the condition in order to benefit the patients.
